# A Multidisciplinary Approach Toward the Anesthetic Management of a Patient With VACTERL Anomaly and Refractory Gastrointestinal Symptoms

**DOI:** 10.7759/cureus.90502

**Published:** 2025-08-19

**Authors:** Noor Sohal, Mamta Chura, Vaibhav Bora, Lindsey Mccabe

**Affiliations:** 1 Anesthesiology, Augusta University Medical College of Georgia, Augusta, USA

**Keywords:** anesthetic management, aspiration risk, bravo capsule, difficult airway, gastric ultrasound, gastroesophageal reflux disease, monitored anesthesia care (mac), tracheoesophageal fistula, tracheoesophageal fistula (tef), vacterl association

## Abstract

Patients with vertebral defects, anal atresia, cardiac defects, tracheo-esophageal fistula, renal anomalies, and limb abnormalities, collectively referred to as VACTERL association, present considerable anesthetic challenges due to the constellation of congenital anomalies that affect multiple organ systems. We report the case of a 27-year-old woman with a complex history of VACTERL association who underwent esophagogastroduodenoscopy (EGD) with Bravo capsule placement for evaluation of chronic nausea and refractory gastroesophageal reflux disease (GERD). Anesthetic considerations included a history of difficult airways, increased aspiration risk, and multiple drug allergies. Preoperative point-of-care gastric ultrasound (POCUS) was employed to confirm an empty stomach, facilitating the decision to proceed with monitored anesthesia care (MAC) while minimizing airway manipulation. This case underscores the importance of multidisciplinary coordination and the integration of bedside ultrasound in optimizing perioperative safety in high-risk syndromic patients.

## Introduction

VACTERL association is a rare, non-random constellation of congenital anomalies that includes vertebral defects, anal atresia, cardiac malformations, tracheoesophageal fistula (TEF), renal anomalies, and limb abnormalities [1,2]. The multisystem involvement characteristic of this condition presents substantial challenges in anesthetic management, particularly during gastrointestinal and airway procedures.

Patients with esophageal anomalies, especially those with a history of surgical repair, such as in cases of TEF or esophageal atresia, are at increased risk of aspiration. This vulnerability stems from altered esophageal motility and impaired gastric emptying [3,4,5]. In such patients, aspiration risk is further amplified by chronic regurgitation, neuromuscular dysfunction, and residual anatomical abnormalities. Preoperative assessment with point-of-care gastric ultrasound (POCUS) has emerged as a valuable, non-invasive tool for evaluating gastric content and guiding anesthetic strategy in high-risk individuals [6,7].

Management strategies to minimize aspiration risk may include using anticholinergic premedication, such as atropine, and rapid sequence induction with neuromuscular blockade using agents like rocuronium. In addition, long-term respiratory sequelae from TEF, such as tracheomalacia, chronic cough, or bronchial hyperreactivity, must be considered when planning anesthetic care. These patients often demonstrate abnormal pulmonary function and are at increased risk of perioperative respiratory compromise [8].

The Bravo pH monitoring system, a wireless capsule affixed to the esophageal mucosa, is commonly used for prolonged evaluation of gastroesophageal reflux. However, the capsule may fail to adhere in patients with prior esophageal surgery or mucosal abnormalities, limiting its efficacy [9]. Awareness of such procedural limitations is essential when managing patients with altered esophageal anatomy.

In this report, we present the anesthetic considerations and procedural challenges encountered in a 27-year-old woman with VACTERL association undergoing esophagogastroduodenoscopy (EGD) with Bravo capsule placement. This case highlights the importance of preoperative gastric assessment, multidisciplinary coordination, and airway risk mitigation in the anesthetic care of patients with complex congenital syndromes. The patient provided written informed consent for both treatment and open-access publication.

## Case presentation

A 27-year-old woman with a known history of VACTERL Association presented for EGD with Bravo capsule placement due to a six-month history of chronic, idiopathic nausea, early satiety, and treatment-refractory gastroesophageal reflux disease (GERD). Her symptoms persisted despite trials of proton pump inhibitors, dietary modifications, and prokinetic agents. She denied any hematemesis, melena, or recent weight loss.

Her past medical history was significant for esophageal atresia repaired in infancy, followed by multiple esophageal dilations and revision procedures. She had complex congenital cardiac anomalies, i.e., ventricular septal defect, micrognathia, and a documented history of complex airway management, including difficult airway (Mallampati Class IV), limited mouth opening, retrognathia, and multiple failed intubation attempts. She also had prior aspiration events. The patient demonstrated preserved functional status with a metabolic equivalent of task (METS) greater than 4, and the anesthesia team classified her as ASA physical status III.

**Figure 1:**
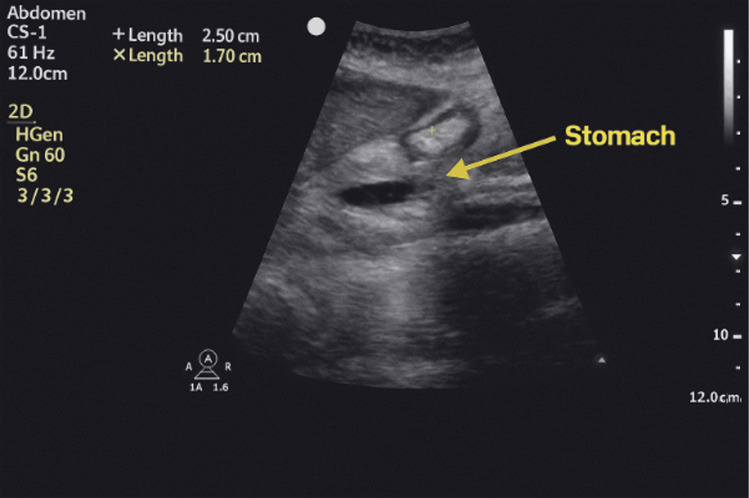
Preoperative gastric ultrasound Arrow indicates the stomach, demonstrating absence of gastric contents.

Given the known history of difficult airway and aspiration, the anesthesia team conducted a POCUS preoperatively, which showed an empty gastric antrum without visible fluid, solid content, or bile (Figure 1). While a gastric ultrasound cannot conclusively exclude small volumes of bile, it remains a valuable risk-stratification tool. The absence of sonographic evidence of gastric content significantly reduced the concern for aspiration, though the team remained prepared for unexpected events, including bile-related chemical pneumonitis.

Despite the presence of multiple risk factors, including difficult airway, prior aspiration, complex cardiac history, and medication allergies, a decision was made to proceed under monitored anesthesia care (MAC) at a mild-to-moderate sedation level, thereby avoiding airway instrumentation. The patient expressed a strong preference for sedation and declined awake fiberoptic intubation. Respecting her wishes, and after detailed discussion of risks and alternatives with the proceduralist and the anesthesia team, a decision was made to proceed under MAC at a mild-to-moderate level of sedation, thereby avoiding airway instrumentation unless clinically indicated. This plan was formulated collaboratively with the proceduralist and the patient, and sedation goals were communicated. Given the anticipated short duration and low-stimulation nature of the procedure, the MAC approach was considered the safest option to minimize airway manipulation.

To mitigate the risk of aspiration and difficult airway, full difficult airway equipment (video laryngoscope, fiberoptic bronchoscope, supraglottic devices, and surgical airway cart) was available at the bedside. Bronchospasm rescue medications were pre-drawn. The team prepared for potential conversion to general anesthesia and for airway support during recovery. ENT was consulted and was available in case tracheostomy is needed. The patient received H₂ blockers, non-opioid antiemetics, and anticholinergic premedication to reduce secretions and aspiration risk.

This case underscores the complexity of anesthetic decision-making in patients with syndromic features and multiple comorbidities. It highlights the necessity of a shared, multidisciplinary approach that respects patient autonomy while maintaining perioperative safety.

Laboratory testing performed prior to the procedure revealed no abnormalities (Table 1). We present a summary of the relevant values below:

**Table 1:** Preoperative laboratory values

Parameter	Result	Reference range
Hemoglobin	13.0 g/dL	12.0-15.5 g/dL
White blood cells	6.5 × 10⁹/L	4.0-11.0 × 10⁹/L
Sodium (Na⁺)	138 mmol/L	135-145 mmol/L
Potassium (K⁺)	3.6 mmol/L	3.5-5.1 mmol/L
Creatinine	0.69 mg/dL	0.6-1.2 mg/dL

Anesthetic and procedural course

The anesthesia team directed management toward preserving spontaneous ventilation and minimizing airway instrumentation, given the patient’s history of difficult intubation, multiple failed intubation attempts, micrognathia, and Mallampati Class IV airway. While awake, fiberoptic intubation was considered a viable option for securing the airway, but the patient declined this approach. Following a thorough multidisciplinary discussion, including consultation with the proceduralist and risk-benefit counseling with the patient, the team proceeded with a carefully titrated MAC plan, with clear thresholds for airway conversion. A difficult airway cart was immediately available at the bedside, equipped with video laryngoscopy, supraglottic airways, fiberoptic bronchoscope, and equipment for surgical airway access. The ENT team was consulted and remained on standby for emergent tracheostomy if needed. Sedation was achieved by the anesthesia team using low-dose midazolam (1 mg IV) for anxiolysis, followed by ketamine and fentanyl bolus titrated slowly to maintain spontaneous ventilation and adequate procedural sedation. Ketamine was selected for its ability to preserve respiratory drive and airway reflexes, although administered in low doses, given the patient’s cardiac history. Propofol was avoided due to the risk of respiratory depression. During the EGD, the endoscopist attempted to deploy the Bravo capsule. However, as in the first attempt, the capsule failed to adhere to the esophageal mucosa and migrated into the hypopharynx, requiring retrieval, but in the second attempt, the procedure was successful. The patient experienced no intraoperative or postoperative complications. She recovered uneventfully, maintained airway reflexes throughout emergence, and was discharged home in stable condition after observation.

## Discussion

In the present case, the patient’s history of tracheoesophageal fistula (TEF) repair, micrognathia, and multiple documented failed intubation attempts presented significant airway management challenges. While awake fiberoptic intubation would be considered the standard of care in a patient with this profile, the patient declined awake intubation and explicitly requested sedation only. Considering this, the anesthesia team conducted a comprehensive risk-benefit assessment, balancing the risks of airway instrumentation under general anesthesia against the risks of proceeding with MAC in a patient with a difficult airway and aspiration history.

MAC was selected with strict procedural criteria, including minimal procedural stimulation, short anticipated duration, ability to maintain spontaneous ventilation, immediate access to advanced airway equipment, ENT surgical backup for emergency airway access, and continuous multidisciplinary communication. While avoiding airway manipulation alone would not typically justify MAC in such a high-risk airway, in this case, the decision was driven by a combination of patient autonomy, risk mitigation strategies, and close procedural coordination. This case highlights the nuanced and individualized decision-making required when balancing standard patients with patient-centered care, especially in syndromic individuals with limited airway options and heightened procedural risk.

The team utilized preoperative POCUS to assess aspiration risk. The scan confirmed an empty stomach, supporting the decision to proceed with MAC while maintaining spontaneous ventilation.

Gastric ultrasound is increasingly integrated into anesthetic workflows, especially for high-risk patients, as it allows real-time assessment of gastric contents and helps inform airway strategy [5,6,10,11,12]. In this case, it served as one of several risk mitigation tools used to balance the patient’s preferences with her complex airway and aspiration risk profile. Additional safety measures included a fully stocked difficult airway cart at bedside, ENT surgical backup pre-notified for emergent tracheostomy, defined conversion criteria to general anesthesia, sedation with low-dose ketamine and fentanyl, and midazolam (1 mg IV) to maintain spontaneous breathing and airway reflexes. These precautions, combined with detailed preoperative briefings, real-time imaging, and pharmacologic planning, ensured a safe and controlled anesthetic course despite the high-risk nature of this case. Lastly, this case exemplifies the critical value of comprehensive, team-based preoperative planning. Syndromic patients, such as those with VACTERL, often require individualized anesthetic strategies, detailed team briefings, emergent airway plans, pharmacologic precautions, and flexible intraoperative decision-making. In our case, these components contributed to a safe and effective perioperative outcome. The case demonstrates that thoughtful integration of bedside diagnostic tools (like gastric ultrasound) with individualized procedural strategy can mitigate risk and improve patient safety, even in highly complex congenital conditions.

## Conclusions

Patients with VACTERL association pose complex perioperative challenges due to their characteristic multisystem anomalies. Effective anesthetic and procedural management in these individuals requires a multidisciplinary, patient-centered approach involving detailed preoperative assessment, individualized airway strategies, and the use of objective tools, such as POCUS, to evaluate aspiration risk.

In this case, the anesthetic plan was formulated through close interdisciplinary collaboration, including advanced coordination with the gastroenterology team, who were aware of the patient's history of a failed capsule placement and the anesthesia team's intent to avoid airway instrumentation. The proceduralist was informed of the limitations of sedation depth under MAC and agreed to proceed only under those parameters. A contingency (Plan B) was also discussed preoperatively, which included an alternative approach in the event of another failed capsule deployment. This case reinforces the importance of team-based planning, shared procedural goals, and coordinated sedation strategies to ensure patient safety, particularly in high-risk syndromic populations. Proactive communication, tailored intraoperative planning, and anticipation of both airway and device-related complications are essential to optimizing outcomes in this vulnerable group.
